# Co-regulatory Network of Oncosuppressor miRNAs and Transcription Factors for Pathology of Human Hepatic Cancer Stem Cells (HCSC)

**DOI:** 10.1038/s41598-019-41978-5

**Published:** 2019-04-03

**Authors:** Rania Hassan Mohamed, Nourhan Abu-Shahba, Marwa Mahmoud, Ahmed M. H. Abdelfattah, Wael Zakaria, Mahmoud ElHefnawi

**Affiliations:** 1 Department of Biochemistry, Faculty of Science, Ain Shams University, Cairo, Egypt; 2 Stem Cell Research Group, Centre of Excellence for Advanced Sciences, Department of Medical Molecular Genetics, National Research Centre, Cairo, Egypt; 3 Department of Mathematics, Faculty of Science, Ain Shams University, Cairo, Egypt; 4 Biomedical informatics and Chemoinformatics group, Centre of Excellence for Advanced Sciences, Informatics and Systems Department, National Research Centre, Cairo, Egypt; 5 Informatics and systems Department, Division of Engineering research, National Research Centre, Cairo, Egypt; 6 Present Address: VAP, CS Department, SUNY, Oswego, NY USA

## Abstract

Hepatic cancer stem cells (HCSCs) are considered as main players for the hepatocellular carcinoma (HCC) initiation, metastasis, drug resistance and recurrence. There is a growing evidence supporting the down-regulated miRNAs in HCSCs as key suppressors for the stemness traits, but still more details are vague about how these miRNAs modulate the HCC development. To uncover some of these miRNA regulatory aspects in HCSC, we compiled 15 down-regulated miRNA and their validated and predicted up-regulated targets in HCSC. The targets were enriched for several cancer cell stemness hallmarks and CSC pre-metastatic niche, which support these miRNAs role in suppression of HCSCs neoplastic transformation. Further, we constructed miRNA-Transcription factor (TF) regulatory networks, which provided new insights on the role of the proposed miRNA-TF co-regulation in the cancer stemness axis and its cross talk with the surrounding microenvironment. Our analysis revealed HCSC important hubs as candidate regulators for targeting hepatic cancer stemness such as, miR-148a, miR-214, E2F family, MYC and SLC7A5. Finally, we proposed a possible model for miRNA and TF co-regulation of HCSC signaling pathways. Our study identified an HCSC signature and set bridges between the reported results to give guide for future validation of HCC therapeutic strategies avoiding drug resistance.

## Introduction

Hepatocellular carcinoma (HCC) is the fourth leading cause of cancer-related death worldwide^1^. Accumulating evidence suggests the hepatic cancer stem cells (HCSC) to be the main organizer for the HCC initiation, as hepatic tumor initiating cells (HTIC). HCSC are a distinct subset of undifferentiated cells endowing tumorigenic and stem-like-characteristics. HCSCs could be identified by various cell surface markers including CD13, CD24, CD44, CD90, CD133, EpCAM (CD326) and OV6^2^, or by selection for the side population cells and those with a high aldehyde dehydrogenase activity^3^. Stemness features of HCSCs include persistent self-renewal, colony and sphere forming abilities and sustained ability of proliferation and differentiation into a tumor bulk. HCSCs are also related to poor outcomes and recurrence in HCC patients, due to their potentials for migration, invasion, metastasis, epithelial-to-mesenchymal transition (EMT) and drug-resistance. Research over the past decade has unraveled that HCSC are regulated by many factors including HCSC niche, genetic and epigenetic microenvironment and stemness-related signaling pathways^2^. These factors drive the CSC to exhibit metabolic flexibility^4^ and promote angiogenesis^5^, neurogenesis^6^ and immune resistance^7^. Moreover, these factors confer the bio-energetic and biosynthetic requirements for maintenance of the tumor homeostasis and progression^2^. Thus, the deeper understanding of the molecular (at genetic and epigenetic levels) properties of this crucial cell population can potentially improve HCC patient outcomes and survival.

Transcription factors (TF) are indispensable players to regulate the cancer stemness pathways. Among these TFs and pathways are Oct4, Sox2, Klf4, and c-Myc, Wnt/β-catenin, IL-6/STAT3, BMI-1, TGF-β, RAS/RAF/MAPK, PI3K/AKT/mTOR, Notch and Hedgehog. Such signaling cascades are serially switched on and off in an alternating and cross-regulated manner in response to environmental variability to maintain the CSC biological and carcinogenic characteristics^2,3^. One of the epigenetic mechanisms, which crucially regulate HCSC hallmarks and hence, their contribution to tumor initiation and drug resistance mechanisms, are micro RNAs (miRNAs). Many reports suggest that single miRNA might target multiple hepatic cancer stemness related signaling pathways by acting as oncogenes or oncosuppressors^8–10^. Also, our group has undergone previous studies to highlight the importance of miRNA in HCC^11–13^, but still more details are hidden about how miRNAs modulate the HCSC mechanisms for HCC development. Tumor suppressor miRNAs, which have been reported to be significantly down-regulated in the HCSC play a key role to inhibit stemness and drug resistance features. Of these miRNAs, miR-145 and miR-148b suppress hepatic cancer stemness via inhibiting Oct4 and neuropilin-1, respectively^14,15^. MiR-199a-3p and miR-148a-3p reduce the drug resistivity in hepatocarcinoma cells by regulating mTOR-c-Met and TGF beta-SMADs, respectively^16,17^.

The previous studies recommended TFs as potential regulatory targets of the dysregulated miRNAs and simultaneously as major gene transcription regulators through binding to the promoter regions of target genes by their DNA-binding domains^18^. TFs and miRNAs are able to co-regulate the expression of targets in forms of feed-forward (FFLs) and feedback loops (FBLs)^19^. The FFL is a motif in which a TF regulates miRNA or miRNA represses a TF, and both of them co-regulate a joint target. FFLs include three types according to the master regulator and regulation of each other: miRNA-FFL, TF-FFL and composite FFL. Regarding the FBL, it is a motif in which a TF and miRNA regulate each other, and each of them regulates their targets individually. Such loops/motifs are important to construct, by means of integrative analysis of transcriptomic data, regulatory networks of gene expression^18^. The resulted Gene Regulatory Networks (GRNs) illustrate the crosstalks between the sets of molecular elements that work together to regulate a biological process and to identify hub elements, which can be recommended as valuable therapeutic targets^20^. An-Yuan Guo group revealed the crucial roles of miRNA-TF co-regulatory networks in Schizophrenia, T-cell acute lymphoblastic leukemia, myocardial infarction and the development of B cell and T cell^21–24^. In our study, we curated the HCSC down-regulated miRNA and their up-regulated gene and TF candidate targets from literature survey, GEO DataSet and the prediction of combined bioinformatics tools. Then, we constructed novel miRNA-TF-gene co-regulatory networks, identified hub elements and proposed a model to link and present a systematic understanding of the molecular mechanisms underlying development of HCSCs and drug resistance. We aim to open new therapeutic strategies to be validated in the future against hepatic cancer stemness and chemoresistance.

## Results

### miRNAs and their targets pathway and gene ontology (GO) enrichment analysis

**Figure 1:**
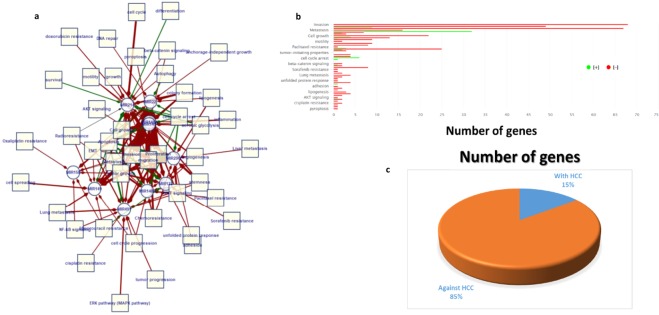
HCC pathogenetic network of the selected HCSC down-regulated miRNAs. (**a**) Network and (**b**) chart are created by miROB online database. Arrows and bars in red indicate the negative effect of the miRNA on the targeted biological process, while green arrows show the positive effect. (**c**) The graph indicates the number of miRNA target genes to promote (with HCC) and to suppress (against HCC) HCC.

**Figure 2:**
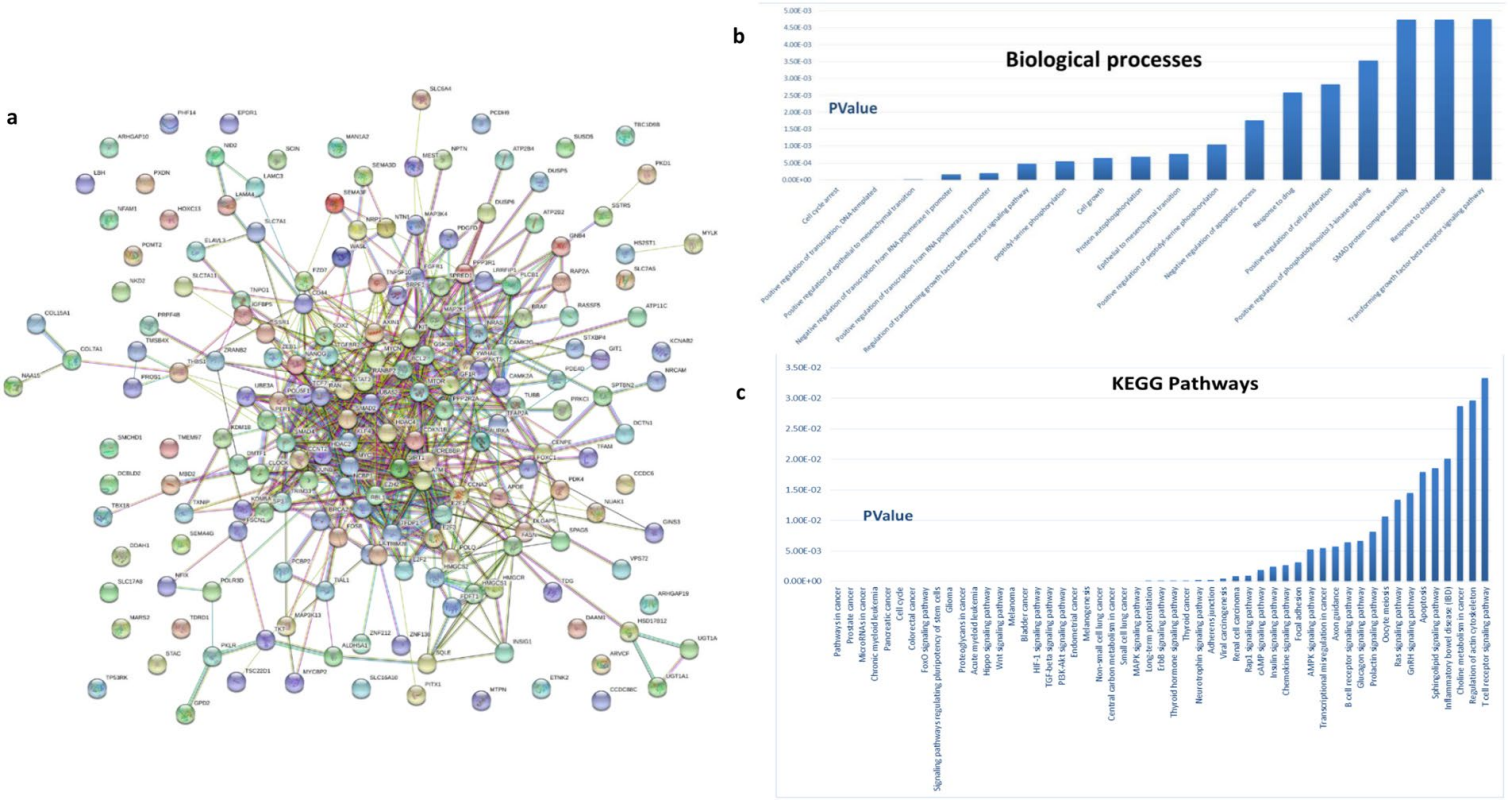
The functional enrichment analysis of the HCSC up-regulated target proteins. (**a**) STRING network. DAVID enrichment analysis represents the involved (**b**) biological processes (GO) and (**c**) pathways (KEGG).

To investigate the oncosuppressor role of the down-regulated miRNA in HCSC development, it was necessary to determine HCSC down-regulated miRNA, up-regulated targets and their functions. We selected the HCSC differentially down-regulated miRNA and up-regulated genes (*P* < *0.05*) through extensive search in publications and databases as described in the Methods section. We curated 13 miRNAs out of the literature mining, and 2 miRNAs from the GEO datasets. Most of the selected 15 significantly down-regulated miRNA in HCSCs are broadly conserved (miR-148a/b-3p, 145-5p, 199a/b-3p, 194-5p, 9-3p, 15b-3p, 22-5p, 122-5p, 214-3p, 29c-3p), except miR-149-5p is intermediately conserved, miR-491-5p is mammalian-specific^25^ and miR-548c-5p is primate-specific^26^. By using miROB intractome online database [mirob.interactome.ru/microRNA_databases], we built a pathogenetic processes network for hepatocellular carcinoma. We found 10 of our 15 selected miRNA (miR-148a/b-3p, 145-5p, 15b-3p, 22-5p, 122-5p, 214-3p, 29c-3p, 149-5p and 491-5p)- the rest are not identified in the miROB intractome database (miR-199a/b-3p, 194-5p, 9-3p and 548c-5p)- are predicted to block/inhibit the expression of high number of genes (85% target genes) involved in the biological processes and pathways of the hepatic cancer stemness and chemoresistance (Fig. 1). That was a first step to proceed forward in our aim and to suggest the role of these miRNAs as hepatic cancer stemness suppressors. We compiled both the validated and predicted targets that are common between our selected bioinformatics target prediction tools and compared them to the significantly up-regulated genes expressed in HCSC and chemoresistant hepatic cancer cells in the literature and GEO database to pick the possible targets to our selected miRNA (Supplementary Table S1). In the target set, we found 31 target genes were originally collected out of the literature mining, and 158 target genes from the GEO datasets. Of these targets, we found around 16% are validated and 84% are predicted targets. Moreover, the STRING analysis showed the high functional connectivity between the targets; 157 of the 189 targets are closely linked together in one cluster (Fig. 2a).

To understand and get more insights for the functional role of the selected miRNA-targets in hepatic cancer stemness, the cancer stemness related pathways (KEGG pathway) enrichment analysis and biological functions (GO) classification of the differentially HCSC-up-regulated targets were performed (Fig. 2b,c). The result revealed that the up-regulated genes were mainly categorized into 53 statistically significant cancer related pathways that form strong associations to cancer stem cell and drug resistance hallmarks (Supplementary Table S2). Also, the top GO terms for biological functions covered the main hallmarks and the pre-metastatic niche of the CSCs including cell cycle arrest, positive regulation of EMT, cell growth, negative regulation of apoptotic process, response to drug and positive regulation of cell proliferation. It is also noteworthy that the most significantly enriched term was the regulation of transcription (Supplementary Table S2). Taken together, our results strongly suggest the selected down regulated miRNAs to be studied as suppressor miRNAs for the main pathways and processes associated with CSCs and drug resistance in liver cancer.

### The HCSC miRNA-TF feed forward and feedback loops (FFLs & FBLs)

Since we found the regulation of transcription is the most enriched term for our targets, we sought to define the target genes controlled by the significantly enriched TFs and to investigate the miRNA-TF mediated FFLs and FBLs among them, hence we can find more clues for regulation of the HCSC. Using RegNetwork software^27^, we got 32 TFs inside the miRNA targets (Supplementary Table S1). Because the miRNA and TF can regulate the same target, we constructed 249 FFLs, of which 157 miRNA-FFLs, 75 TF-FFLs and 17 composite-FFLs (Supplementary Table S3), proving that in our system, the miRNA-FFLs were the prominent networks regulating the HCSC development. By testing the significance of the miRNA-FFLs via the randomization test using FANMOD software^28^, we found that they are highly significant (*P* ≤ *0.001*), indicating the strong relativity of the selected miRNAs, TFs and target genes in the HCSC and drug resistance characteristics. We could not construct more than two verified HCSCs miRNA-FFLs networks using the verified regulations (yellow highlighted rows in miRNA-FFL table; Supplementary Table S3), which suggests the novelty of the recommended new regulatory mechanisms included in our miRNA-FFL networks. Moreover, as the miRNA and TF can also regulate each other we also could construct 4 novel feedback loops.

### The HCSC miRNA-TF co-regulatory network

**Figure 3:**
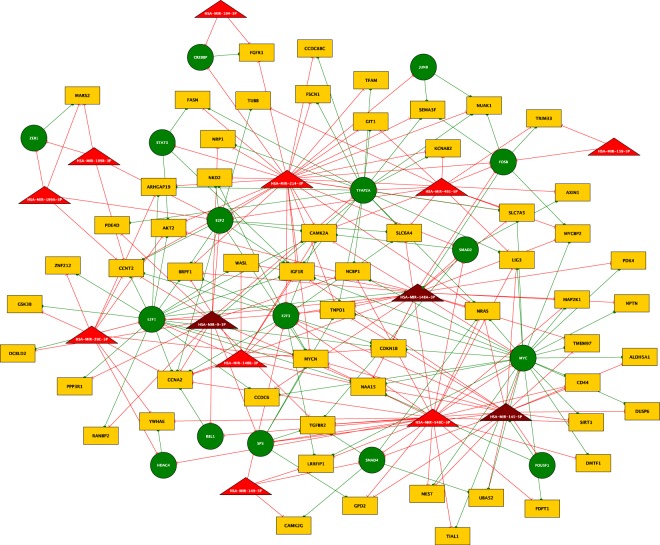
The proposed co-regulatory network of HCSC. The network created by MATLAB. Green circles are TFs, yellow rectangles are target genes, red triangles are miRNA, and purple triangles are miRNA acting also as TF target genes. Red edges are directed from miRNA, while green edges directed from TF to their targets.

HCSC miRNA-TF co-regulatory networks are effective way to study the hepatic cancer stemness gene regulation. To construct a co-regulatory network of HCSC and drug resistance development, we merged the FBLs and the miRNA-FFLs. Thirteen HCSC-miRNAs (87% of the selected miRNA), 16 HCSC-TFs (50% of the targeted TFs) and 58 HCSC- target genes formed 87 nodes and 266 edges in our network (Fig. 3). In this co-regulatory network, miR-214 and TFAP2A form the largest sub-network to co-regulate many genes (14 miRNA-FFLs) and both have verified association with HCSC and CSC development^29,30^. Some of these correlations are consistent with the experimentally verified ones, such as, miR-214 roles to repress TFAP2A, NRAS and FSCN1^31,32^. Moreover, IGF1R, TNPO1 and FASN are experimentally validated targets for TFAP2A, as annotated by RegNetwork software, and are involved in multiple cancer stemness and drug resistance processes^30,33–35^.

### miRNA-TF sub-networks role in regulating HCSC

**Figure 4:**
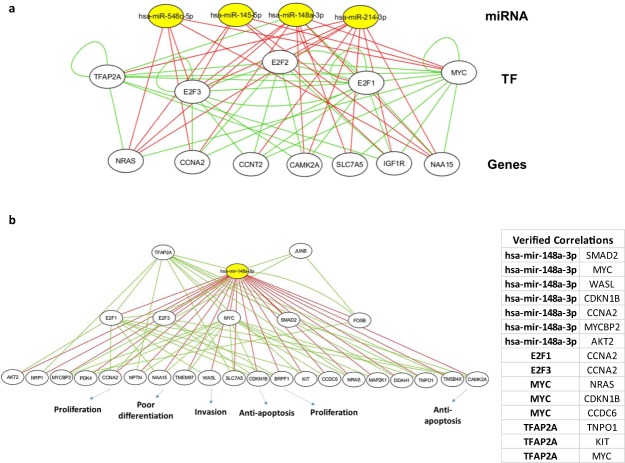
Sub-networks extracted from the HCSC miRNA and TF regulatory relationships. (**a**) Sub-network among the representative hubs. (**b**) The miR-148a sub-network model. Red edges are directed from miRNA, while green edges directed from TF to their targets.

For better understanding of the HCSC regulation and interpreting the reliability of our regulatory network, hubs have been selected according to the criteria mentioned in the Methods. We selected 9 regulators including 4 miRNA hubs (miR-214, miR-548c, miR-145 and miR-148a) and 5 TF hubs (MYC, TFAP2A, E2F1, E2F2 and E2F3) and 7 target gene hubs (SLC7A5, CAMKIIA, NAA15, NRAS, CCNA2, IGF1R and CCNT2). All the hubs were found to be strongly connected as shown in the extracted hub sub-network (Fig. 4a). MYC here is a very rich hub regulated by 3 miRNA and 9 TFs, while it regulates 2 miRNA and 43 genes of the HCSC up-regulated targets. Moreover, it links to 5 of the 7 hub genes. Out of the hub sub-network, we found E2F3 has been shown to be the most regulated gene by 6 hub elements (4 miRNA and 2 TFs), while it regulates 4 hub elements (3 genes and a TF). Of the hub miRNAs, miR-148a is the most promising one to be regulated by 2 hub TFs and regulates 8 hub elements (3 TFs and 5 genes). The hub genes here are related to many enriched biological processes and pathways supporting the cancer stemness and drug resistance traits such as anti-apoptosis, proliferation, poor differentiation and EMT (Supplementary Table S2).

It is noteworthy to mention that, the gene having the highest number of in-miRNA-TF common edges in our network is SLC7A5, which is related to essential amino acid (EAA) intake and confirms the importance of the EAAs metabolism for maintaining the hepatic cancer stem cell phenotype^36,37^. This is also confirmed here with the significant enrichment of SLC7A5 in the central carbon metabolism in cancer, which has essential role in CSC metabolic niche^35^. We here suggest a possible direct correlation between SLC7A5 and glutamine metabolism on hepatic cancer stemness which may be supported by Liao *et al*. They have reported that, the deprivation of glutamine inhibits the glutathione synthesis and disrupts the redox balance of the CSC. This increases the reactive oxygen species (ROS) level, which reduces the stemness characteristics and drug resistance by degradation of β-catenin and decrement of ABCG2 expression, respectively^37^. Also, it has been reported that, the increase of ROS levels can lead to CSC apoptosis^38^.

Moreover, it has been reported that, although its function remains to be elucidated, the overexpression of NAA15 is strongly related with the cancer poor differentiation. The homology of NAA15 to yeast N-acetyltransferase (NAT) 1 may indicate its function in protein acetylation, which has great impact on cellular differentiation, growth and apoptosis^39^. This has been also confirmed by involvement of NAA15 into our GO enriched biological processes of positive regulation of transcription and negative regulation of apoptotic process. The expression level of the cell cycle regulators (i.e. CCNT2 and CCNA2) has been linked to cell cycle progression and cancer growth. CCNT2 is a member of cyclin family that facilitate the transcription activity of RNA polymerase II by regulation of cyclin-dependent kinase (CDK) kinase during the cell cycle. Moreover, in the gastrointestinal tract tumors, the bile acid receptor FXR dependent suppression of cancer cell proliferation has been suggested to be through suppression of CCNA2^40,41^. In addition to sharing of IGF1R and CAMKIIA in HIF-1 signaling pathway in our enriched cancer stemness pathways, high IGF1R and CAMKIIA expression enhances the carcinogenesis by inducing the stemness, chemoresistance and anti-apoptosis. It has been reported that, IGF1R induces drug-resistivity of Huh7 against anti-cancer agent, through inhibition of caspase-3. Also, CAMKIIA has anti-apoptotic effect through activation of NFκB, which in turn activates the sarco/endoplasmic reticulum Ca2^+^-ATPase (SERCA) to enhance the survival of CSC under the metabolic stress^33,42^. This result suggests the hubs to have important controlling effects through different regulatory roles in HCSC development. So, we are here suggesting several novel routes of hepatic cancer stemness and drug resistance regulation dependent on our hub genes.

The previous result attracted our interest to study the significance of our network by checking the FFL and FBL sub-network of the miR-148a regulation in the HCSC (Fig. 4b). Several studies identified the role of miR-148a in pathogenic processes related to the CSC such as, chemoresistivity and EMT, which suggest it as a therapeutic tool for hepatic cancer stemness^17,43^. By extending the bridges of our sub-network with the literature survey results, we confirmed the oncosuppressor model of miR-148a through inhibition of cancer stemness by targeting SMAD2 in HCSC, AKT2 and KLF4. SMAD2 is a key player for the TGF-β signaling pathway in the initiation of the hepatic cancer, where it induces the EMT and CSC-like properties and markers. MiR-148a has been shown to inhibit cell proliferation and EMT properties in bladder cancer through ERBB3/AKT2/c-myc and ERBB3/AKT2/Snail signaling. KLF4 inhibition was shown to suppress the cyto-protective autophagy process, which is considered as a source of energy for the anti-apoptotic gastric cancer cells^17,43,44^. Also, we proposed miR-148a targeting more genes with oncogenic effects such as, NAA15, IGF1R, SLC7A5, CAMKIIA, CCNT2 and NRAS, etc. In addition, we proposed that TFAP2A, FOSB, E2F1 and JUNB may regulate the expression of miR-148a. These results and links confirm the significance of our networks and suggest more therapeutic roles for up-regulation of miR-148a to suppress hepatic cancer stemness through targeting many processes such as cell cycle progression, drug resistance, HCSC redox and metabolic balance.

## Discussion

Hepatic cancer stem cells are strongly considered as the tumor initiator and main players for the cancer relapse and chemoresistance^2^. This study opened a window on the roles of the selected down-regulated miRNAs and their predicted and validated targets in the hallmarks of the hepatic cancer stemness development and drug resistivity. Moreover, as we found many TFs as miRNA targets, we evaluated the co-regulation of the miRNAs and TFs for the HCSC related up-regulated genes. We revealed that, the different means of this co-regulation may control many significant pathways in HCSC development. For the first time, we constructed novel cancer stemness miRNA-TF FFL networks to uncover new regulatory mechanisms. We also proposed new bridges linking up many published results about these important hepatic cancer stemness and drug resistivity pathways. So that, we widen the scope to understand more roles for the selected miRNA over the experimentally validated ones and shed the light over the mechanisms involved in the HCSC development.

In agreement with the previous reports and reviews, our results proved the significant roles of the selected miRNAs on suppressing the pathways and biological processes associated with the cancer stemness such as, cell cycle, apoptosis, adherence junction, EMT and Wnt, TGF-β, PI3K-Akt, MAPK, ERBB and neurotrophin signaling pathways^8,9,29,43^. Also, we found many signaling pathways regulating cancer stemness that can be controlled/targeted by our selected miRNA panel such as, FoxO, HIF-1, and Hippo signaling pathways. The PI3K/Akt/mTOR signaling pathway activation could induce stemness traits through decreasing the ROS levels. The PI3K/Akt/mTOR signaling pathway activates nuclear localization of FoxOs and stimulates the hypoxia-inducible factor- 1α (HIF-1α), which in turn stimulates the transcription of FoxOs, that regulate the catalase production and ROS removal^45^. This agrees with the ongoing wave of research in oncology that the antioxidant defense is fundamental for maintenance of stemness and drug resistance in cancer cells^45,46^. In addition to their roles in the maintenance of redox balance inside the CSCs, FoxOs transcription factors translocation into the cytoplasm prevents the expression of death receptor ligands to enhance the survival of the cancer cells^47^. The CSCs-like properties of EMT and chemoresistance are also maintained via HIF-1α activation of, not only FoxOs, but also SIRT1 production mediated by NFκB pathway^48^. Target genes such as those involved in the Wnt and TGF-β pathways can inhibit Hippo pathway and accumulate YAP/TAZ proteins. This consequently induces EMT stimulated by TGF-β pathway and drug resistance via different mechanisms such as, actin remodeling or connective tissue growth factor (CTGF) production^49^. These results suggest our selected miRNAs may overcome the resistance of the CSC to the known drug targets by inhibiting more signaling pathways involved in the development of hepatic cancer stemness.

Our analysis also showed the proposed great impact of the selected miRNAs to act as oncosuppressors and control some novel significantly enriched pathways, which do/might enhance the crosstalk between the HCSC and its microenvironment. These pathways include the pathways regulated by hormonal and metabolic changes (i.e. Proteoglycans in cancer, Choline metabolism in cancer, Sphingolipid signaling pathway, Prolactin signaling pathway, Thyroid hormone signaling pathway, Glucagon signaling pathway, Insulin signaling pathway and Central carbon metabolism in cancer), neurogenic (i.e. Axon guidance) and immunological pathways (i.e. T cell receptor signaling pathway, Inflammatory bowel disease (IBD), B cell receptor signaling pathway and Chemokine signaling pathway) and pathways featuring the stem cell pluripotancy (i.e. Signaling pathways regulating pluripotency of stem cells and Oocyte meiosis). Moreover, there are many enriched pathways linked to the development of other cancers, which proposes the significant links of the selected miRNA to inhibit, may be in the same way, the development of many other tumors.

We found that many of the HCSC-miRNA targets are enriched significantly as transcription factors and the other targets are mostly common targets to the miRNAs and TFs in consistence with the concept of Cui *et al*.^50^. The previous result drove us to construct the FFLs and FBLs for investigation of the co-regulatory mechanisms of HCSC miRNA-TF. We found the extracted hub elements of these networks (Fig. 4a) are highly connected and reported to have experimentally validated important roles in cancer stemness hallmarks development. For instance and in accordance with Chen *et al*. and Khan *et al*., who defined E2F family emerging roles in EMT and cancer cell proliferation via cell cycle progression^51,52^. We found here 3 members of E2F family as hub elements for hepatic cancer stemness promotion. Not only the hub elements, but it is noteworthy that the extracted subnetworks such as the miR-214 and TFAP2A may shed the light on the importance of the sumoylation pathway in maintenance of the hepatic cancer stemness. Small ubiquitin-like modifier (SUMO)-conjugated TFAP2A transcription factor induces the expression of CD44, which maintain the cancer stemness characteristics of breast and colorectal CSCs^30^. Moreover, the miR-148a subnetwork showed interesting results that linked several pathways in pathology of HCSC. The resulted networks could work as a bridge for the previously reported results and considered as a step for more studies to experimentally validate these links.

**Figure 5:**
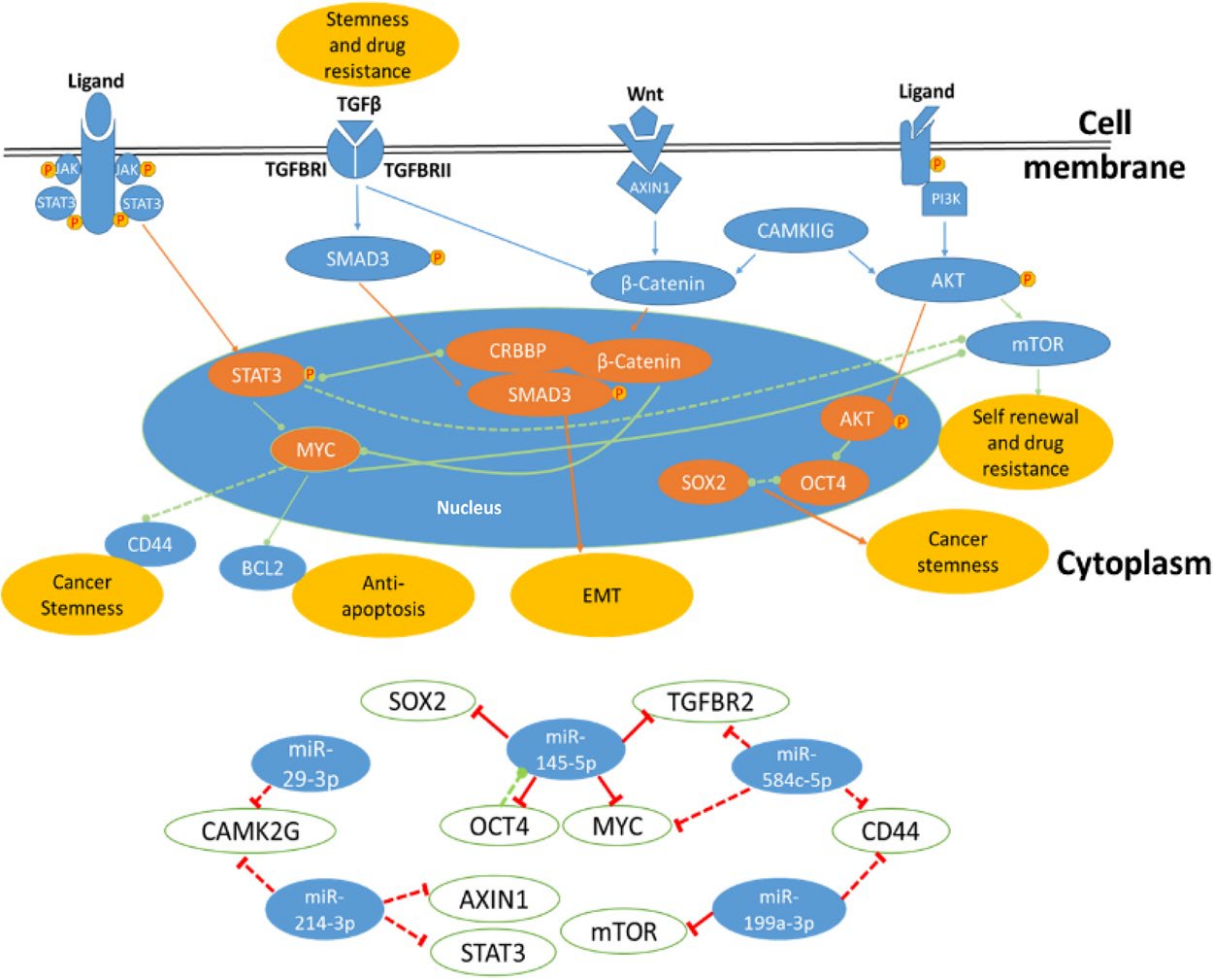
The proposed model of the interacted HCSC pathways regulated by the selected miRNAs and TFs. Solid lines are the experimentally validated, while dashed lines are the predicted correlations. Red lines are directed from miRNA, while green lines directed from TF to their targets.

Here, we also proposed a partial model (Fig. 5) based on the links we found in our study, which may illustrate the possible crosstalk between several pathways such as, JAK/STAT, Wnt, TGF-β, PI3K signaling pathways involved in the HCSC pathology and possible blocking of these pathways through our suppressor miRNA panel. Studies showed that TGF-β regulates the cancer stem cell self-renewal and differentiation properties via inducing leukemia inhibitory factor (LIF) and IL-11 activation of the JAK/STAT pathway and STAT3 phosphorylation in glioblastoma and colon cancer, respectively. Also, inhibition of STAT3 activation was shown to reduce cancer stemness and sphere formation. Blocking of Wnt signaling pathway has been reported to inhibit Wnt dependent gene expression of the stemness markers, EMT, metastasis and sphere formation. Moreover, the PTEN, an inhibitor for the PI3K signaling pathway, represses the expression of stemness and drug resistance markers OCT4, SOX2, NANOG and MDR1 in glioblastoma. mTOR is considered a main player of the stemness and drug resistivity induced by PI3K pathway^16,53–55^. Here and as a result of linking the reported results and our predicted correlations, the model is partially proposing the crosstalk of all these pathways to enhance the hepatic cancer stemness in means of: 1- MYC and STAT3 may connect JAK/STAT, Wnt and PI3K in HCSC to activate expression of CD44 stemness marker, mTOR as a self-renewal and drug resistance inducer and inhibit apoptosis via activation of Bcl2. We also showed a significant enrichment of metabolic, hormonal and immunological pathways that might enhance the microenvironment of the HCSC dependent on MYC and/or STAT3 such as, Proteoglycans in cancer, Prolactin signaling pathway, Thyroid hormone signaling pathway, Central carbon metabolism in cancer, Inflammatory bowel disease (IBD) and Chemokine signaling pathway (Supplementary Table S2). 2- Smad3, β-catenin and CRBBP complex may connect TGF-β and Wnt pathways in HCSC to induce EMT through high expression level of α-smooth muscle actin (α-SMA). 3- CAMKIIG may increase hepatic cancer stemness characteristics by inducing the expression of MYC in β-catenin dependent manner and stemness markers, Oct4 and SOX2, expression via induction of AKT phosphorylation. Also, our functional enrichment analysis suggested CAMKIIG to regulate the HCSC microenvironment through regulation of Proteoglycans in cancer, Neurotrophin signaling pathways, Glucagon signaling pathway and Oocyte meiosis pathway (Supplementary Table S2).

The limitation of this study might be that, 1- Some of the targets are shown to be validated according to DIANA-TarBase, but we found the way of validation is indirect or unknown and, then included these targets as predicted. 2- The number of the HCSC oncosuppressor miRNA may be more than our selected miRNA, but we tried to collect the most confirmed down-regulated ones. 3- Other limitation, some of the collected significantly up-regulated targets in this study were shown in the literature to be liver oncosuppressors such as, AXIN1^56^. One probability is that the up-regulated gene is mutated^57^ or has a role in drug resistivity, but not directly in stemness characteristics. So that, our study suggests more experimental and mutational studies to prove the predicted roles of such genes in liver cancer stem/resistant cells.

In conclusion, we could link up miRNA, TF and their targets in networks to support more understanding of the HCSC regulation and to guide more researches for validation of these links. Also, our results and proposed model may suggest therapeutic strategies by targeting the enriched pathways via the selected oncosuppressor miRNA panel. Inspiring from the hub elements and the functionally enriched pathways in our study, we are confirming some recommended and suggesting new therapeutic targets for inhibition of these enriched pathways. For example, 1- Targeting MYC protein, which regulates cell cycle and drug resistivity represented here in controlling CCNT2 and IGF1R, respectively. MYC is also considered as a linker between several signaling pathways to maintain the liver cancer stem cell phenotype, microenvironment and drug resistance as shown in our results and others^58^. 2- Targeting E2F family, especially E2F3, aiming for cell cycle arrest and anti-proliferative effect on the HCSC^41,51^. 3- Targeting of SLC7A5 protein to inhibit the essential amino acid uptake and metabolism, hence inhibiting the metabolic niche and induction of the oxidative stress in cancer stem cells^36,37^. (4) Targeting CAMKIIG, which might inhibit several pathways regulating different aspects in HCSC properties and microenvironment.

## Methods

### Collection of HCSC down-regulated miRNAs

To collect a set of significantly down-regulated miRNAs in HCSCs, we performed an extensive literature mining, in order to search for studies that detected the significantly down-regulated miRNAs (*P* < *0.05*) in HCSCs. These miRNAs were derived from HCC patients, two or more hepatic cancer stem-like cell lines, or at least one hepatic cancer stem-like cell line, but the miRNAs derived from a single hepatic CSC-like cell line was reported to have a role against hepatic cancer stemness. We excluded the down-regulated miRNAs with reported dual role in cancer stem cell development. We used PUBMED search tool using the keywords “(hepatic cancer stem cells OR drug resistant hepatic cancer stem cells) AND (miRNAs)^8,29,59,60^”. We also searched using Gene Expression Omnibus (GEO) database [ncbi.nlm.nih.gov/geo/] with the same keywords for screening a large deal of functional genomic and epigenomic studies and found the GEO dataset of accession number GSE66529. PhenomiR knowledgebase [tools4mirs.org/software/mirna_databases/phenomir/] was also searched for confirming the selected microRNA expression in HCC and their related biological processes. The identified set of miRNAs was entered to miRBase [mirbase.org] to get the accession numbers of their mature forms.

### MicroRNA target genes prediction and identification

Firstly, microRNA target analysis was done using miRWalk 2.0 server [mirwalk.uni-hd.de/]. MiRWalk is a comprehensive database from which both predicted and experimentally validated miRNA-target could be obtained^61^. MiRWalk target prediction server provides miRNA targets obtained from the intersection of different prediction algorithms. In our analysis, we selected the target prediction algorithms: miRWalk, Targetscan v7.0, miRanda, RNA22, Mirmap, and Pictar to obtain the common predicted targets with cut off *p*-value < 0.05. Also, to widen our human target search, we added the targets predicted by miRanda [microrna.org] using the filter of (view target sites with all miRNAs with good mirSVR scores) and confirmed the binding site by RNA22 [cm.jefferson.edu/rna22/] and/or intaRNA [http://rna.informatik.uni-freiburg.de/IntaRNA/Input.jsp]. Secondly, we collected the significantly up-regulated HCSC genes (*P* < *0.05*) from GEO datasets of GEO accession numbers GSE73571, GSE59713 and GSE47932 and from the literature mining in PUBMED search tool using keywards (“hepatic cancer stem cells OR drug resistant hepatic cancer stem cells) AND (genes OR gene expression profile OR gene expression array”)^59,62–67^. We curated the up-regulated target genes from data derived from HCC patients or two or more hepatic cancer stem-like cell lines. Then, we selected only those target genes up-regulated in HCSC and drug resistant hepatic cancer cells, by comparing the common targets coming from the target prediction tools with those HCSC up-regulated targets got from the literature mining and GEO datasets. Both predicted and validated targets of each selected miRNA were merged together in a target list, and then integrated to undergo subsequent enrichment analysis.

### Functional annotation enrichment analysis and pathogenic network inference

Functional enrichment analysis for the obtained miRNA targets was performed using DAVID server [Database for Annotation, Visualization and Integrated Discovery], [david.ncifcrf.gov], which provides the most enriched gene ontology (GO) terms as well as pathways from several databases such as KEGG, Biocarta, Reactome and others offering the most relevant functions in which a certain gene list can be involved^68^. The most essential enriched gene ontology annotations and pathways, which are extracted from DAVID with Bonferroni correction and false discovery rate (FDR) correction for multiple testing by filtration P value < 0.05 in responses to the hepatic cancer stemness and chemoresistance, were then deeply studied. To predict the proposed role of the selected miRNA, we used miROB database [mirob.interactome.ru/microRNA_databases] and built a pathogenetic processes network for hepatocellular carcinoma using the selected set of miRNAs as an input. In addition, STRING network database [string-db.org] was used to obtain functional protein interaction networks between our miRNA targets.

### Prediction of regulatory interactions between miRNA, target genes and TFs

We used the targets of our miRNA set that act as transcription factors (TF) to explore the miRNA-TF-Gene regulatory interactions. We could obtain the combinatorial regulatory interrelations between miRNAs, TFs and genes using RegNetwork database [regnetworkweb.org]^27^. RegNetwork collects a list of TFs for human and mouse from FANTOM, UniProt, TRANSFAC and JASPAR. RegNetwork provides the experimentally validated, as well as the predicted transcriptional and post-transcriptional regulatory interactions using KEGG, TRED, TRANSFAC and JASPAR databases depending on the transcription factor binding sites (TFBS). The TFBS conservation data in RegNetwork database were collected from the UCSC Genome Browser and Ensembl databases. The RegNetwork confirms the conserved binding sites by computing a score not less than the threshold score for the data provided from UCSC Genome Browser, which are calculated by TFLOC program and LiftOver tool of UCSC, and by documenting the alignment information of Ensemble database using MOODS software^27^.

### Generation of Feed forward (FFL) and feedback loops (FBL) and statistical analysis

**Figure 6:**
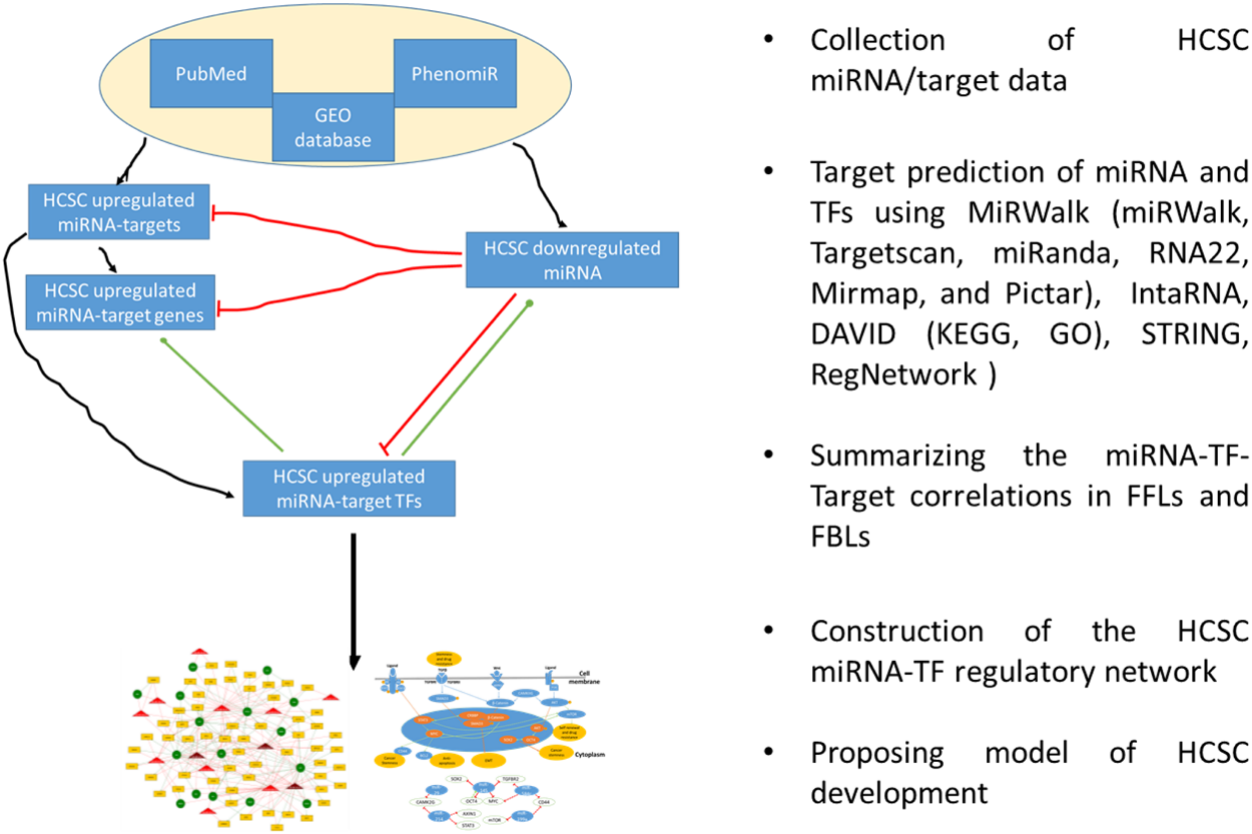
The flow chart illustrating the construction of the HCSC co-regulatory network.

We summarized the miRNA, TF and gene interactions and constructed miRNA-FFL, TF-FFL, Composite-FFL and FBL manually as shown in the Supplementary Table S3; sheet 2–5 according to the flow chart in Fig. 6. Then, we tested the significance of miRNA-FFL by running random permutation using FANMOD tool. Only motif found more than 5 times of Z-score higher than 2 and *p*-value < 0.05 in FANMOD export settings was considered significantly enriched. According to the program manual, firstly, we converted each element in the miRNA-FFL into number manually and arranged them as binary correlations (edges), each per line in a notepad.txt file (i.e. in the first miRNA-FFL between hsa-mir-148a-3p, E2F1 and CCDC6, the hsa-mir-148a-3p, E2F1 and CCDC6 are enumerated as 1, 2 and 3, respectively), so their arrangement were as following:

1 “tab” 2

1 “tab” 3

2 “tab” 3

Then, we selected this file within the input graph frame and chose the network to be directed. The algorithm options were selected to be full enumeration and the subgraph size of 3. In the randomization test, the motifs are detected by comparing their frequency of occurrence in the original network to their frequency of occurrence in a number of similar, yet randomized networks. We used 10,000 random networks with the default settings of local constant number of bidirectional edges and edge exchange parameters (3 exchanges per edges and 3 tries per exchange). The *p*-value was set to be the number of random networks in which the motif occurred more often than in the original network, divided by the total number of random networks.

### HCSC co-regulatory network, sub-networks and hub analysis

To construct a proposed HCSC co-regulatory network, we merged the miRNA-FFL and FBL, then we implemented two computer programs to process and visualize the resulting network elements (nodes and edges). Briefly, we developed a computer program using the Matlab® programming environment [Matlab© release 2017a, The MathWorks, Inc., Natick, Massachusetts, United States] to summarize the relationships among pairs of miRNAs, TFs, and Genes from the input dataset shown in the Supplementary Table S3; sheet 6. The relationships corresponding to these pairs can intuitively be visualized as “from X to Y”, where X and Y are the pairs in a relationship. We labeled the whole input dataset as *Reg*. The dataset, *Reg*, is represented in this program as an “*N* by 3” matrix (i.e., a matrix with *N* rows and three columns; Supplementary Table S3; sheet 6). Based on this *Reg* matrix, our program implementation extracted all kinds of the required relationships as output formalized by defining the following two set-theoretic categorizations (called R and G) of equations (1) and (2):12

Our hand-coded programs are implemented in a way that discovers and deletes duplicates, so that the visualization neither repeats the same nodes nor repeats the same edges between the same nodes. As a second step, the summarized relationships resulting from our hand-coded computer program were used as an input to a general-purpose diagramming application [yWorks GmbH (2018). yEd Graph Editor [software: r.3.18], retrieved from yworks.com/yed], which we used to visualize the whole network in a graph-like structure, with the miRNAs, TFs, and Genes being the graph nodes, while the relationships among the corresponding elements being the graph edges. We then extracted the hub elements (miRNA, TF or gene) from the different correlations according to the following rules modified from^21–24^: 1- The summation of the out and in edges of the miRNAs and TFs are higher than the average. 2- The targets must be common targets for miRNAs and TFs, and the in edges are equal or higher than 10. Then, we focused on several sub-networks and hub sub-networks, which were visualized using Cytoscape version 2.8^69^.

## Supplementary information

Supplementary datasets legends

Dataset 1

Dataset 2

Dataset 3
