# Supplementary material for: Co-regulatory Network of Oncosuppressor miRNAs and Transcription Factors for Pathology of Human Hepatic Cancer Stem Cells (HCSC)
Source: Sci Rep. 2019 Apr 3;9:5564. doi: 10.1038/s41598-019-41978-5 (PMC6447552; doi:10.1038/s41598-019-41978-5)
Supplement: Supplementary file 1 — Supplementary datasets legends [file 41598_2019_41978_MOESM1_ESM.docx]

**Co-regulatory Network of Oncosuppressor miRNAs and Transcription Factors for Pathology of Human Hepatic Cancer Stem Cells (HCSC)**

Rania Hassan Mohamed^1^, Nourhan Abu-Shahba^2^, Marwa Mahmoud^2^, Ahmed M. H. Abdelfattah^3^**^±^**, Wael Zakaria^3^, Mahmoud ElHefnawi^4,5*^

^1^Department of Biochemistry, Faculty of Science, Ain Shams University, Cairo, Egypt.

^2^Stem Cells Research Group, Centre of Excellence for Advanced Sciences, Department of Medical Molecular Genetics, National Research Centre, Cairo, Egypt

^3^Department of Mathematics, Faculty of Science, Ain Shams University, Cairo, Egypt.

^±^ Current Address: VAP, CS Department, SUNY Oswego, NY, USA.

^4^Biomedical informatics and Chemoinformatics group, Centre of Excellence for Advanced Sciences, Informatics and Systems Department, National Research Centre, Cairo, Egypt.

^5^Bioinformatics group, Centre of excellence for medical research, National Research Centre, Cairo, Egypt.

* Correspondence: Prof. Mahmoud ElHefnawi, mahef@aucegypt.edu

**Supplementary table legends:**

Supplementary Table S1: The target genes of the selected down-regulated miRNA and their target TFs in HCSC.

Supplementary Table S2: The functional enrichment analysis of the HCSC miRNA-up-regulated target genes (KEGG, GO)

Supplementary Table S3: The FFLs, FBL and miRNA-TF network co-regulating HCSC pathways.
